# Segmentation Method for Magnetic Resonance-Guided High-Intensity Focused Ultrasound Therapy Planning

**DOI:** 10.1155/2017/5703216

**Published:** 2017-06-22

**Authors:** A. Vargas-Olivares, O. Navarro-Hinojosa, M. Maqueo-Vicencio, L. Curiel, M. Alencastre-Miranda, J. E. Chong-Quero

**Affiliations:** ^1^Tecnologico de Monterrey, Campus Estado de México, Atizapán de Zaragoza, MEX, Mexico; ^2^Tecnologico de Monterrey, Campus Santa Fe, Álvaro Obregón, Ciudad de México, Mexico; ^3^Electrical Engineering Department, Lakehead University, Thunder Bay, ON, Canada

## Abstract

High-intensity focused ultrasound (HIFU) is a minimally invasive therapy modality in which ultrasound beams are concentrated at a focal region, producing a rise of temperature and selective ablation within the focal volume and leaving surrounding tissues intact. HIFU has been proposed for the safe ablation of both malignant and benign tissues and as an agent for drug delivery. Magnetic resonance imaging (MRI) has been proposed as guidance and monitoring method for the therapy. The identification of regions of interest is a crucial procedure in HIFU therapy planning. This procedure is performed in the MR images. The purpose of the present research work is to implement a time-efficient and functional segmentation scheme, based on the watershed segmentation algorithm, for the MR images used for the HIFU therapy planning. The achievement of a segmentation process with functional results is feasible, but preliminary image processing steps are required in order to define the markers for the segmentation algorithm. Moreover, the segmentation scheme is applied in parallel to an MR image data set through the use of a thread pool, achieving a near real-time execution and making a contribution to solve the time-consuming problem of the HIFU therapy planning.

## 1. Introduction

**Figure 1:**
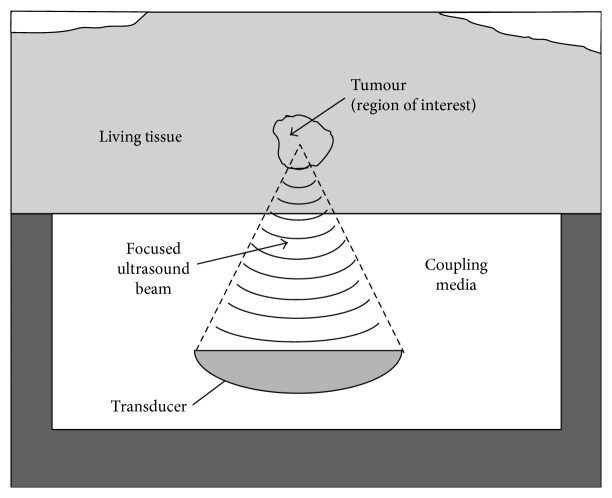
Objects in the HIFU therapy.

High-intensity focused ultrasound (HIFU) is a minimally invasive therapy modality in which ultrasound beams are concentrated at a focal region, producing a rise of temperature and selective ablation within the focal volume and leaving surrounding tissues intact [1]. HIFU has been proposed for the safe ablation of both malignant and benign tissues and as an agent for drug delivery [2]. Magnetic resonance imaging (MRI) has been proposed for guidance and monitoring for the therapy as it provides anatomical images with an adequate spatial resolution. On the other hand, MRI is sensitive to temperature changes [3]. The combination of HIFU and MRI is known as magnetic resonance-guided HIFU (MRgHIFU). The objects that are found in a HIFU treatment include the ultrasonic transducer, acoustic coupling medium (such as water, oil, and gel pads), and the tissue to be treated as shown in Figure 1.

In MRgHIFU therapy planning, the identification of regions of interest, such as regions within the tissue and around the transducer, is performed in order to define the target tissue region and the transducer position along with the localization of its geometric focus. Several MR images are used to cover the volume of interest to be treated. Once the position of the geometric focus (focal point) is obtained, it is guided towards the target tissue [4]. If a proper identification is achieved, it would be possible to calculate in advance the effects of the application of the therapy, given the current distribution of regions of interest. Image segmentation algorithms have been proposed as an alternative to the manual identification of the regions of interest, a time-consuming problem in the therapy planning [5]. This problem becomes more noticeable if several images are required for the therapy planning. Different image segmentation techniques can be used for the identification of regions. The watershed transform [6] is a popular segmentation method in medical imaging [7]. Segmentation of MR images, with solutions based on the watershed segmentation algorithm, has been proposed before in other studies. In [7], an improvement to the watershed transform that enables the introduction of prior information in its calculation, in the form of markers generated from atlases, was presented. With the additional information, they limited the oversegmentation that occurs when segmenting medical images. The authors applied the algorithm to knee cartilage segmentation and white matter/gray matter segmentation in MR images, while demonstrating similar or superior performance to that of manual segmentation by experts, at an average of 0.94. To get a precise liver segmentation in abdominal MR images, Masoumi et al. [8] proposed an algorithm that utilizes MLP neural networks to extract features of the liver region to be used with the watershed algorithm. The extracted features are used to monitor the quality of the segmentation using the watershed transform and adjust the required parameters automatically. The average accuracy they achieved was 0.94 while running faster than other methods. Similar approaches have been used in [9, 10], to segment regions in brain and breast images in order to help with medical diagnosis, specifically cancer for the latter.

The fast execution of an image segmentation task has been an object of particular interest and has been addressed before in Shasidhar et al. [11] performing modifications in the standard segmentation algorithm and in Rowińska and Gocławski [12] using graphics processing units (GPUs) as an alternative to improve the execution of the segmentation algorithm. Both approaches work with the fuzzy c-means (FCM) algorithm. Approaches that consider different segmentation algorithms could be implemented.

The purpose of the present research work is to implement an efficient segmentation scheme for the MR images used for the HIFU therapy planning. In addition, it is intended that the implementation works in near real-time (i.e., that the segmented images are available after a time delay introduced by the segmentation process itself [13]) in order to address the time-consuming problem of the therapy planning. The segmentation scheme is based on the watershed method for the identification of the regions that are found on the HIFU treatment. Since the segmentation process is intended to be performed on a large amount of MR images, a thread pool was implemented in order to take advantage of all the available CPU cores for processing. This reduced the processing time of the group of MR images, but not the processing time of the watershed segmentation algorithm. The employed MR images of the present research work were obtained from a study of the distribution of heat during abscess treatment in a murine model where the transducer was positioned vis-à-vis the desired target [14].

## 2. Materials and Methods

**Figure 2:**
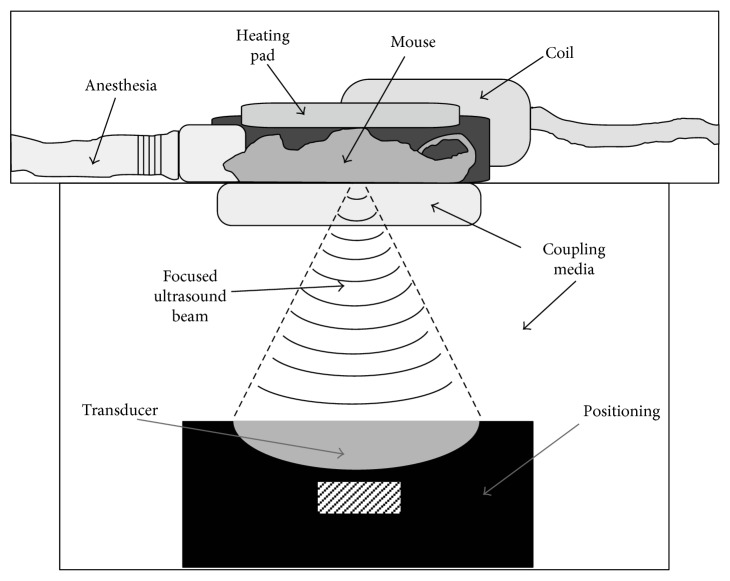
The experimental setup for abscess treatment in mice with focused ultrasound guided by MRI [14].

An experimental protocol for the modeling of the thermal effects of the ultrasound is proposed. T1-weighted MR images were obtained from a study of the distribution of heat during abscess treatment in a murine model using a 3T MRI scanner (Achieva, Philips Healthcare). The transducer was positioned vis-à-vis the desired target. The protocol for this study was approved by the Lakehead University Animal Care Committee. The setup for the study is shown in Figure 2.

### 2.1. Magnetic Resonance Images

**Figure 3:**
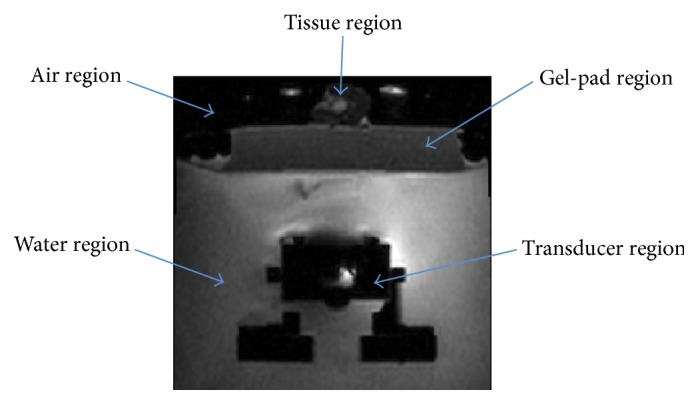
Main regions in the MR image (transverse image).

Transverse and sagittal T1-weighted MR images were obtained with a 3T MRI scanner (Achieva, Philips Healthcare). The field-of-view (FOV) is 120 × 120 × 48 mm. Voxel size is 0.5 mm, and the slice thickness is 2 mm [14]. Intensity inhomogeneity is present in the MR images. The regions to be identified are air, tissue, gel-pad, water, and transducer as shown in Figure 3.

### 2.2. Watershed Segmentation Algorithm for Image Segmentation

The watershed algorithm is based on visualizing an image in three dimensions: two spatial coordinates and an intensity value. In the algorithm, three categories of points are considered: points that belong to a regional minimum, points at which a hypothetical drop of water, if placed at the location of any of those points, would fall with certainty to a single minimum and points at which water would equally fall to more than one minimum. For a given regional minimum, the set of points satisfying the second condition is called “catchment basin.” The points satisfying the third condition form crest lines on the surface and are known as “watershed lines.” In watershed segmentation, a common application is the extraction of nearly uniform objects from the background [15].

#### 2.2.1. The Use of Markers in Watershed Segmentation Algorithm

If the watershed segmentation algorithm is applied directly to the image, oversegmentation will be obtained due to irregularities in the image, yielding a useless result [7, 15]. Oversegmentation is controlled by means of markers, as a tool that brings additional knowledge to the segmentation algorithm. For the generation of markers, two main steps should be considered: preprocessing and definition of criteria that markers must fulfill [15].

For the proposed segmentation scheme, during preprocessing, noise is removed using a Gaussian filter. Then, five separate markers were defined for the watershed segmentation algorithm: three internal (tissue, gel-pad, and transducer) and two external (air and water). All the markers were stored in a separate image, each with a different value (so that the watershed algorithm could differentiate each one) and a black background.

**Figure 4:**
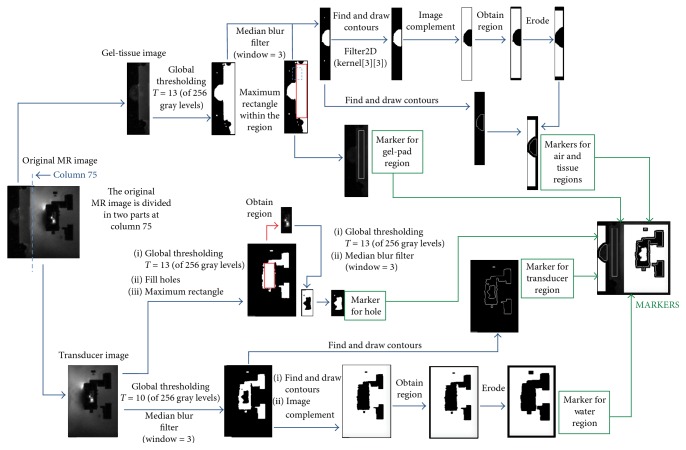
Definition of markers for the watershed segmentation algorithm. The image with the required markers to perform the segmentation is labeled as “MARKERS.”

The overall process to select the markers for the watershed segmentation algorithm is shown in Figure 4.

#### 2.2.2. Definition of the Gel-Pad and Tissue Region Markers

The first step was to separate the air, gel-pad, and tissue from the transducer and the water. By reviewing the images in all of the groups, it was identified that the gel-pad and tissue are always present before the column 75 of the images and that the tissue is always smaller than the gel-pad. This information was used to create the markers for the air, tissue and gel-pad from the left image (henceforth called gel-tissue image), and the marker for the transducer and water from the right image (henceforth called transducer image).

The gel-pad region was inscribed as a rectangular region. An algorithm to find the largest rectangle submatrix on a binary matrix [16] was used on the gel-tissue image. The gel-tissue image was binarized using an intensity of 15 in a grayscale of 256 gray levels, and, since the gel-pad was larger than the tissue, the maximum rectangle found in the image was inscribed in the gel-pad.

To obtain the marker for the tissue region, the gel-tissue image was divided taking the column that corresponds to the upper left corner of the gel-pad marker as frontier. A binary matrix was obtained using a threshold of 13. A median blur filter, with a blur window of 3, was used in order to improve the shape of the tissue contours. From the resulting binary image, the contours of the tissue region were obtained with the “findContours” function in OpevCV [17]. If more than one contour was present, the one with the largest area was selected as the tissue marker.

#### 2.2.3. Definition of the Air Region Marker

The tissue marker was used as the base for the air marker. It was filled with a different value than the air marker, and then a convolution using the kernel {{1, 1, 1}, {1, 0, 1}, and {1, 1, 1}} was performed until there were no black pixels within the tissue. The air region resulted from the negative mask of the convoluted tissue region. The resulting binary image was eroded two times in order to have a separation between the marker of the air region and the marker of the tissue region.

#### 2.2.4. Definition of the Transducer Region Marker

A binary threshold with an intensity of 10 was applied to the transducer image that was previously obtained. Then, a median filter was used to remove any noise surrounding the transducer region. Finally, the contours of the image were found with the “findContours,” “approxPolyDP,” and “drawContours” functions. An additional marker was needed for the transducer object: the hole inside it. To obtain it, the largest rectangle was found within the transducer marker, and the area inside of the rectangle was segmented and thresholded with an intensity of 13. All the black pixels were converted to the hole's value, and all white pixels were turned into black so that only the hole remained. Finally, a median filter was used to remove any pixels that were not part of the transducer hole.

#### 2.2.5. Definition of the Water Region Marker

The transducer marker was used as the base for the water marker. The complement of the transducer marker was obtained and was consequently eroded twice so that there existed a separation of the water and transducer regions.

### 2.3. Processing of Image Groups

**Figure 5:**
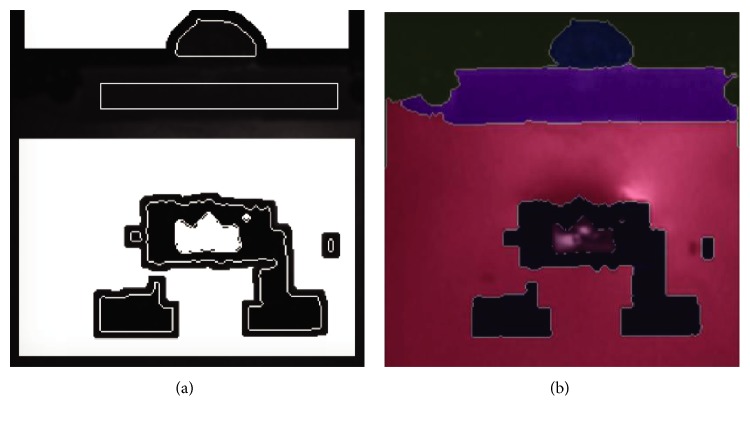
Application of the watershed segmentation algorithm with markers. (a) Image containing all the generated markers. (b) Segmentation of the regions of interest with the watershed algorithm.

Once all the markers are generated, a marker image that contains them was created. A sample of all the markers in a single image can be seen in Figure 5(a). That marker image can be used with the watershed algorithm to segment the five regions of interest. The result of a segmentation of a sample image can be seen in Figure 5(b).

The segmentation has to be performed on a group (data set) of 224 MR images. In order to achieve a near real-time processing, a thread pool was used to apply the proposed solution to the data set in parallel.

In order to carry out the thread pool tests, the data set was segmented using the watershed segmentation algorithm (with markers). A Hewlett-Packard (HP) Z420 workstation with a 4x Intel® Xeon® CPU E5-1607 v2 @3.00GHz processor and 8GB RAM was used for the segmentation. The processor has four cores, and the operating system in this workstation is Ubuntu 14.04.1 LTS. The segmentation of the data set is done with two experiments: using one CPU core in the thread pool and using four CPU cores in the thread pool.

## 3. Results

F-measure was considered for the evaluation of the image segmentation quality [18]. This evaluation method has been used for the evaluation of the segmentation quality in previous research work [4].

**Figure 6:**
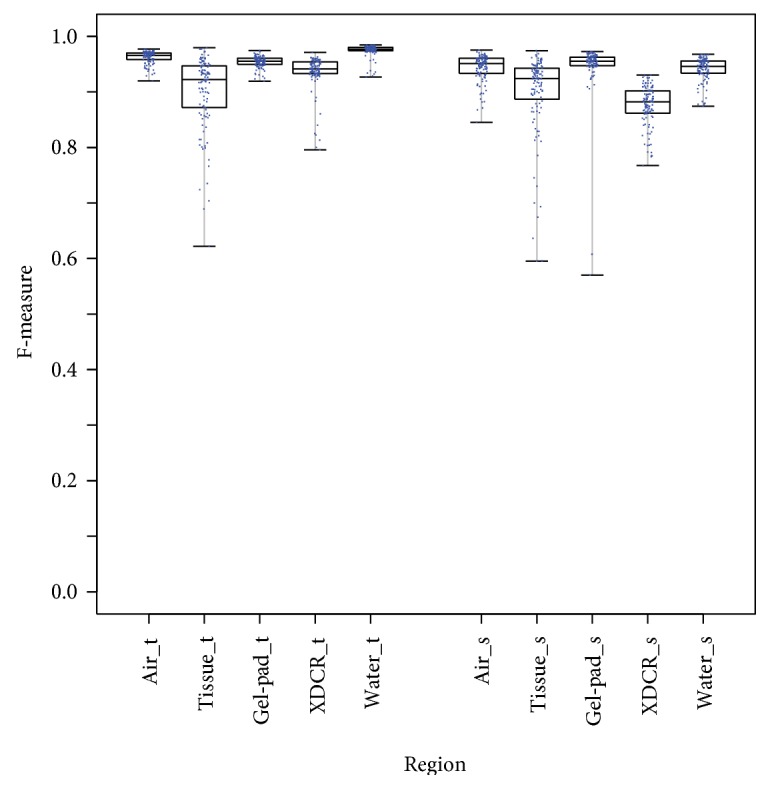
Watershed segmentation using markers in each region of interest. The letters “t” and “s” stand for transverse and sagittal planes, respectively. This graph was generated with R [19].

Segmentation of the five regions of interest (air, tissue, gel-pad, transducer, and water regions) was performed with the watershed segmentation algorithm using markers over the group of MR images. The obtained results, evaluated with F-measure, are shown in the set of boxplots in Figure 6. In the figure, the air, tissue, gel-pad, transducer, and water regions are labeled as “Air,” “Tissue,” “Gel-Pad,” “XDCR,” and “Water,” respectively.

**Figure 7:**
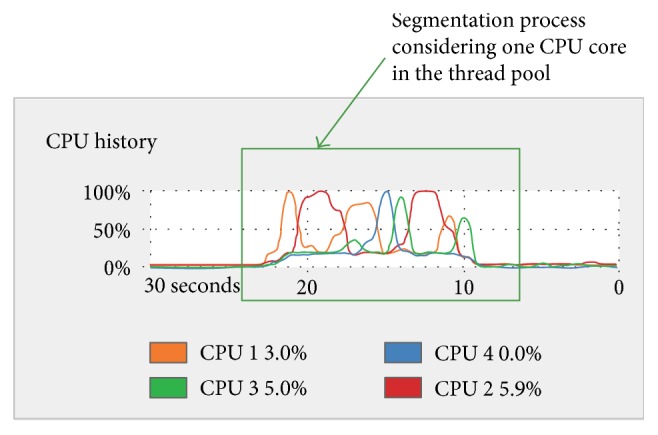
Segmentation process considering one CPU core in the thread pool displayed in the system monitor of the HP Z420 workstation. For a group of 224 images, the total execution time was 11.8811 sec.

**Figure 8:**
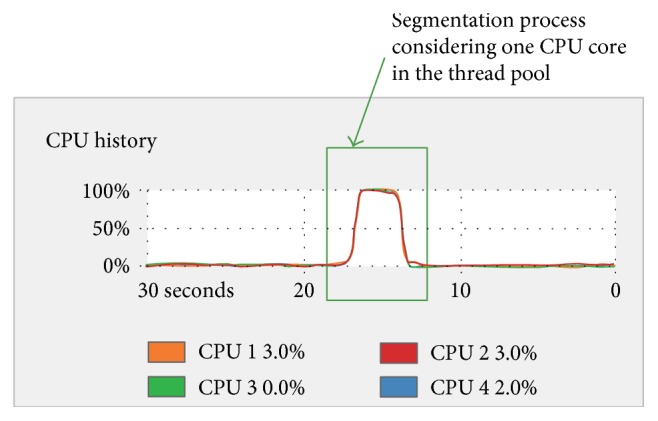
Segmentation process considering one CPU core in the thread pool displayed in the system monitor of the HP Z420 workstation. For a group of 224 images, the total execution time was 3.0682 sec.

When the segmentation was performed with one thread in the thread pool, the segmentation process was carried out using all the CPU cores available at different times as shown in Figure 7, resulting in an average execution time of 11.8811 sec. When the segmentation was performed with four threads in the thread pool, as shown in Figure 8, the execution time was 3.0682 sec.

## 4. Discussion

The use of watershed segmentation algorithm with markers yielded results that were evaluated with F-measure with medians above 0.8 for each region as shown in Figure 6.

The use of watershed segmentation algorithm with markers yielded results that were evaluated with F-measure. For the air region, the obtained medians were 0.9657 and 0.9510 in transverse and sagittal images, respectively. For the transducer regions the obtained medians were 0.9416 and 0.8821 in transverse and sagittal images, respectively. For the water region, the obtained medians were 0.9769 and 0.9459 in transverse and sagittal images, respectively. In the case of the tissue region, the obtained minimum values were 0.6219 and 0.5954 in transverse and sagittal images, respectively. Despite this fact, the results as a group yielded medians of 0.9224 and 0.9241 in transverse and sagittal images, respectively. On the other hand, for the gel-pad region, the obtained minimum value was 0.5701 in sagittal images. Despite this fact, the results as a group yielded a median of 0.9553 in this case.

From the thread pool results shown in Figures 7 and 8, it can be appreciated that in both cases, the CPU is using all its cores to perform a given segmentation task. However, the configuration of the thread pool considering the maximum number of cores (in this case, the use of four cores) makes the CPU work with its resources at the maximum capacity and at the same time. This results in the performance of the task in less time than using the configuration of one single core in the thread pool. An average speedup of 3.8723 in comparison with the version that only uses a single CPU core was obtained. This average speedup could be increased further if more CPU cores were available.

## 5. Conclusions

The implementation of the watershed segmentation algorithm with markers was carried out to address the problem of segmentation of MR images for the HIFU therapy planning. The achievement of the best possible identification of objects in the MR images was sought through the previous knowledge of the image features in order to generate proper markers for the watershed segmentation algorithm. Moreover, it was intended to achieve a time-efficient segmentation process when working with image groups.

The achievement of a functional segmentation process is feasible, but the preliminary image processing steps are required in order to define the markers for the segmentation algorithm. Despite the presence of intensity inhomogeneity in the employed MR images, the use of segmentation methods especially designed to address this problem was not necessary at all because the watershed segmentation algorithm with markers proved to have enough capacity to deal with this problem, still yielding functional segmentation results.

In order to improve the segmentation accuracy, the generation of the markers could be reevaluated. Currently, the markers are generated with information obtained from previous observation of the images. However, as can be seen in Figure 6, there were some cases in which the segmentation accuracy was really low, at around 50%. Using techniques such as neural networks [8] to generate the markers could help improve the segmentation accuracy.

By using a thread pool to apply the segmentation scheme to all the MR images of a given data set, a near real-time execution was achieved. This represents an additional contribution to solve the time-consuming problem of the HIFU therapy planning.

Another alternative that could be considered to reduce execution time is parallel processing with graphics processing units (GPUs). However, there were some limitations with the data set, primarily that the images are too small, so a redefinition of the proposed solution in order to align it with a GPU scheme is required.
